# Metabonomic profiles delineate potential role of glutamate-glutamine cycle in *db/db* mice with diabetes-associated cognitive decline

**DOI:** 10.1186/s13041-016-0223-5

**Published:** 2016-04-18

**Authors:** Yongquan Zheng, Yunjun Yang, Baijun Dong, Hong Zheng, Xiaodong Lin, Yao Du, Xiaokun Li, Liangcai Zhao, Hongchang Gao

**Affiliations:** Radiology Department of the First Affiliated Hospital, Wenzhou Medical University, Wenzhou, 325027 Zhejiang China; School of Pharmaceutical Sciences, Wenzhou Medical University, Wenzhou, 325035 Zhejiang China; Department of Urology, Renji Hospital, Shanghai Jiao Tong University School of Medicine, Shanghai, 200127 China

**Keywords:** Diabetes-associated cognition decline, Nuclear magnetic resonance, Metabonomics, Glutamate-glutamine cycle

## Abstract

**Background:**

Diabetes-associated cognition decline is one of central nervous system complications in diabetic mellitus, while its pathogenic mechanism remains unclear. In this study, ^1^H nuclear magnetic resonance-based metabonomics and immunohistochemistry was used to explore key metabolic alterations in hippocampus of type 2 diabetic *db/db* mice with cognition decline in order to advance understanding of mechanisms underlying the pathogenesis of the disease.

**Results:**

Metabonomics reveals that lactate level was significantly increased in hippocampus of *db/db* mice with cognition decline compared with age-matched wild-type mice. Several tricarboxylic acid cycle intermediates including succinate and citrate were reduced in hippocampus of *db/db* mice with cognition decline. Moreover, an increase in glutamine level and a decrease in glutamate and γ-aminobutyric acid levels were observed in *db/db* mice. Results from immunohistochemistry analysis show that glutamine synthetase was increased and glutaminase and glutamate decarboxylase were decreased in *db/db* mice.

**Conclusions:**

Our results suggest that the development of diabetes-associated cognition decline in *db/db* mice is most likely implicated in a reduction in energy metabolism and a disturbance of glutamate-glutamine shuttling between neurons and astrocytes in hippocampus.

**Electronic supplementary material:**

The online version of this article (doi:10.1186/s13041-016-0223-5) contains supplementary material, which is available to authorized users.

## Background

Type 2 diabetes is a chronic and metabolic disease characterized by hyperglycemia due to insulin resistance and β-cell dysfunction [1, 2]. Its resultant complications are becoming main public health problems. Diabetes-associated cognitive decline (DACD) as a central nervous systems complication in type 2 diabetes has already been attracted considerable attention, not only for its negative effect on the brain but also its association with other neurodegenerative diseases [3–5]. DACD has been recognized in diabetic patients [6] and animal models [1, 4, 7]. Hence, it is of great interest and importance to explore the underlying mechanisms and develop treatment strategies for the disease.

Metabolic and vascular disturbances have been found to be implicated in the pathophysiology of cognitive impairment in diabetes [3]. Hyperglycemia can affect morphology of the neuron in the hippocampus and in turn impair learning and memory [8, 9]. Artola et al. found that the inhibition of long-term potentiation and facilitation of long-term depression in the hippocampus may contribute to learning and memory deficits associated with diabetes. Moreover, hippocampal neurons have been shown to undergo apoptotic cell death under hyperglycemic conditions [10], while expression of the astrocyte-specific glial fibrillary acidic protein (GFAP) is enhanced [11]. Also alterations in dendritic spine length and density were reported in diabetic animal models [12]. So far, many factors have been found to influence the development of DACD, such as brain vasculature, glucose toxicity, oxidative stress, hypoglycemic episodes, and glucose metabolism dysregulation. However, metabolic alterations associated with the disease need to be further explored.

Nuclear magnetic resonance (NMR)-based metabonomic analysis as a systems biology approach aims to detect the global metabolic information in biological samples [13, 14]. It has been extensively applied in the diagnosis and evaluation of diabetes and the provision of crucial insights into the pathogenesis of diabetes [15, 16]. By using ex vivo ^13^C NMR approach with glucose and acetate as substrate, we found enhanced pyruvate recycling pathway in earlier stage of type 1 diabetic rats induced by streptozotocin, and disordered metabolic trafficking between the astrocyte and neuron in later stage, which may be a regulated mechanism against metabolic impairments [17]. In addition, we also found hypoglycemia induced by insulin can affect different metabolic pathways, such as neurotransmitter transition, energy metabolism, and other metabolic equilibrium in a brain region-dependent manner [18]. These results contribute to the understanding of the underlying mechanisms that leads to brain damages in type 1 diabetes. However, the global metabolic changes in brain tissue of type 2 diabetes are not well understood.

To comprehensively profile the metabolic changes associated with the development of DACD, type 2 diabetes *db/db* mice were used and developed features of cognitive decline with age. NMR-based metabonomics with key protein analysis was performed to study the characteristics of metabolism in the hippocampal samples obtained from db/db mice with DACD. Therefore, the aims of the present study were: (1) to investigate behavioral changes in *db/db* mice with DACD, and (2) to explore metabolic variations in hippocampus using NMR-based metabonomics. The results will advance understanding of potential mechanisms underlying DACD.

## Results

### Learning and memory performance

**Fig. 1:**
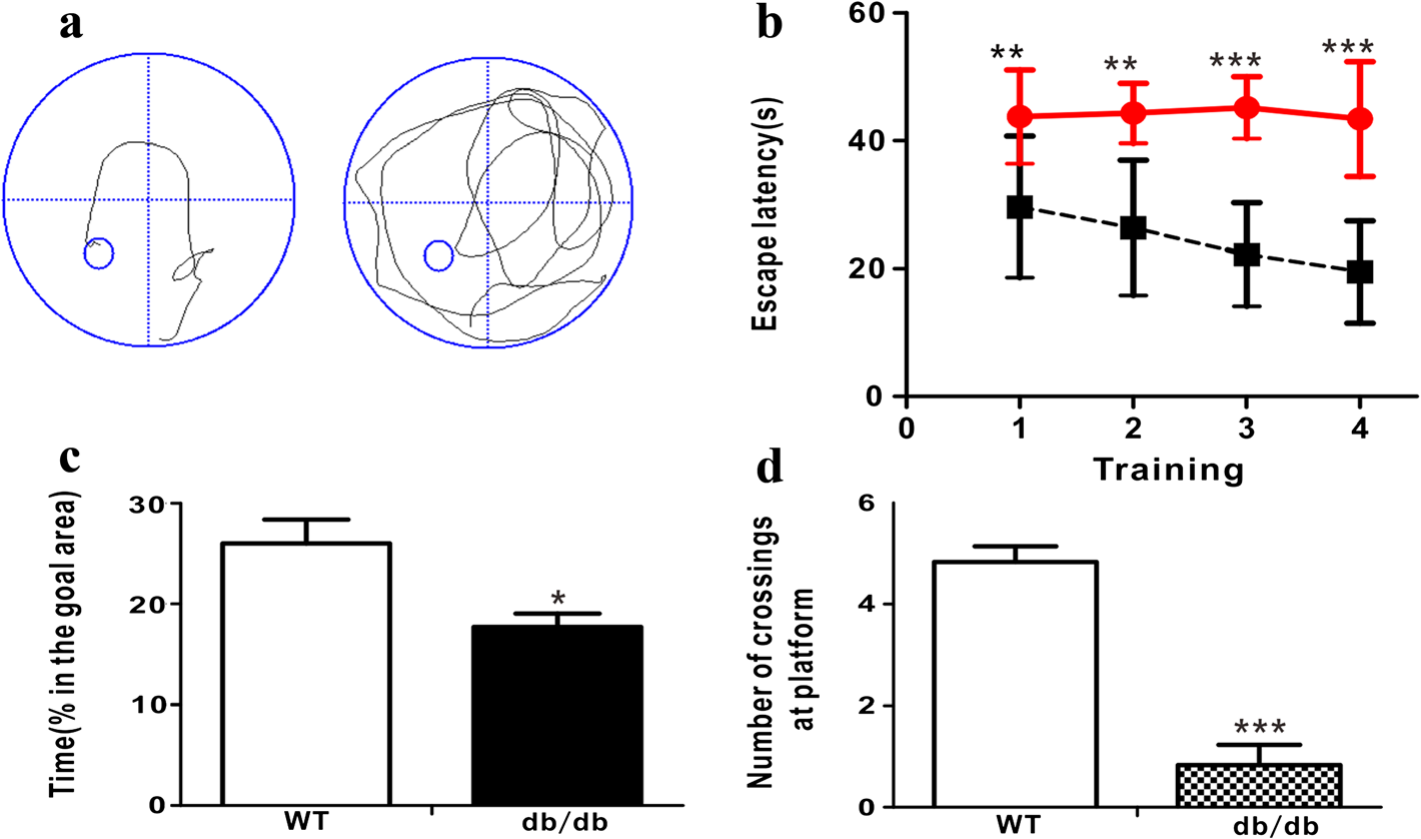
Performance of Morris water maze test in *db/db* (*n* = 7) and WT mice (*n* = 7). **a** Swim track; (**b**) Escape latency; (**c**) Time spent in goal area; (**d**) Number of crossing the original platform. Significant level: ^*^ *P* < 0.05, ^**^ *P* < 0.01, ^***^ *P* < 0.001

The Morris water maze (MWM) test showed that escape latency of *db/db* mice was significantly longer than that of age-matched wild type (WT, Fig. 1a and b) mice. In addition, *db/db* mice had a significantly shorter swimming time and crossing number as compared with the age-matched WT mice in the target quadrant during the probe trial in the MWM test (Fig. 1c and d). Thus, results of the test indicate learning and memory deficits in *db/db* mice at 17-wk of age.

### Histopathological examination in hippocampus tissues

**Fig. 2:**
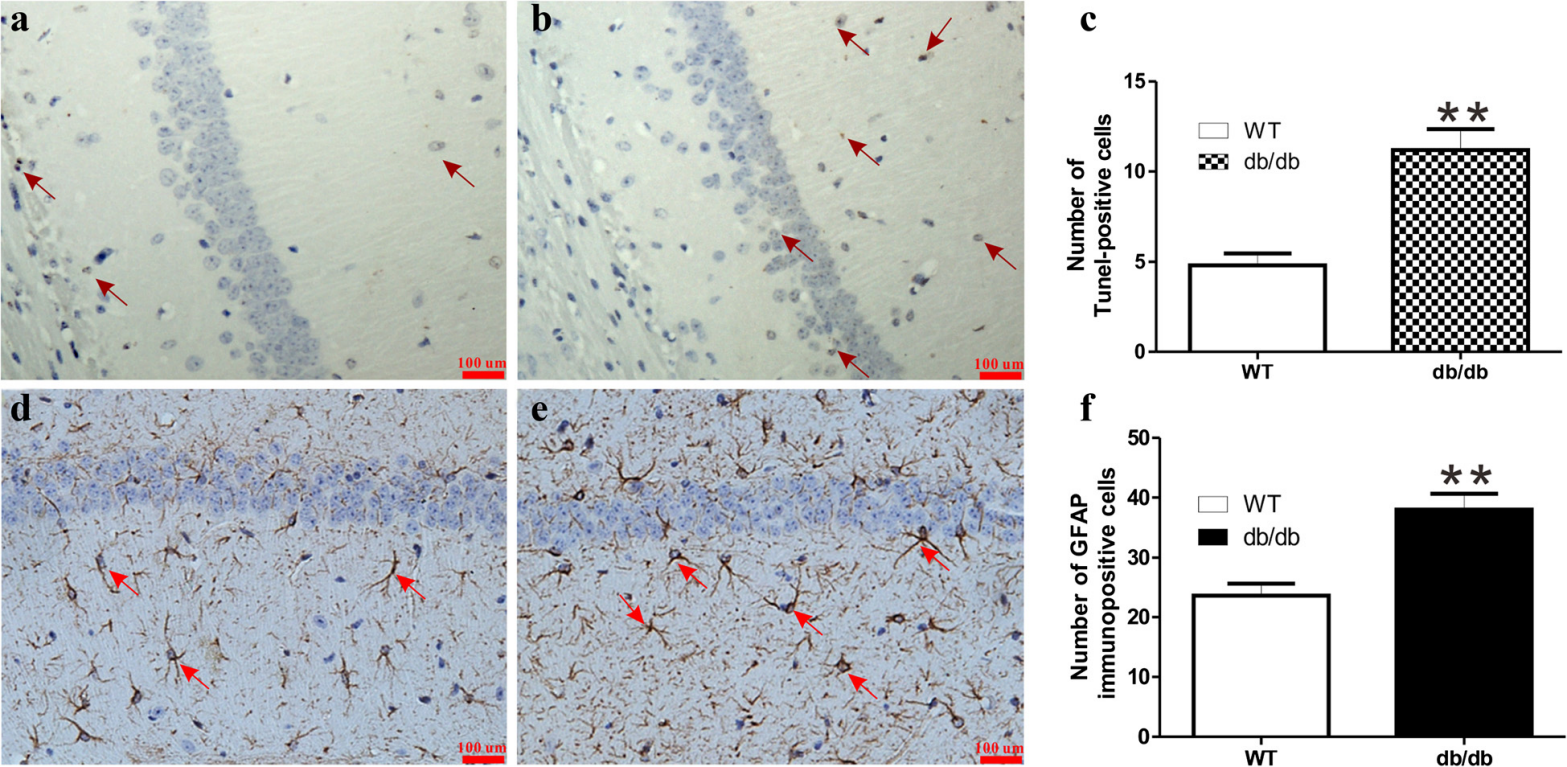
Pathological changes in hippocampus of *db/db* and WT mice. Apoptosis was examined by the TUNEL assay ((**a**), WT mice; (**b**), *db/db* mice with cognitive decline). Astrogliosis was evaluated by the GFAP method ((**d**), WT mice; (**e**), *db/db* mice). The numbers of TUNEL and GFAP-positive cells were counted blindly, as shown in (**c** and **F**), and an average was taken from five different fields of hippocampus in each group of mice (*n* = 4, Scale bars = 100 μm.). Significant level: **P < 0.01

Figure 2 illustrates the histological changes in hippocampus based on TUNEL assay and GFAP immunohistochemistry. It can be seen that number of TUNEL-positive cells were significantly increased in hippocampus of *db/db* mice with DACD compared to WT mice (Fig. 2a-c, *P* < 0.01), which suggests that there was more neuronal apoptosis in hippocampus of db/db mice. Moreover, relative to WT mice, the expression of GFAP, which is a key indicator of astrocyte reactivity, was increased significantly in *db/db* mice with DACD (Fig. 2d–f, *P* < 0.01).

### Metabonomic analysis of the hippocampus extracts

**Fig. 3:**
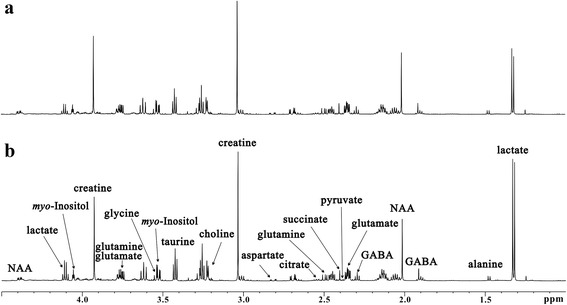
Representative 600 MHz ^1^H NMR spectra of hippocampus extracts from WT mice (**a**, *n* = 11) and *db/db* mice (**b**, *n* = 7)

**Fig. 4:**
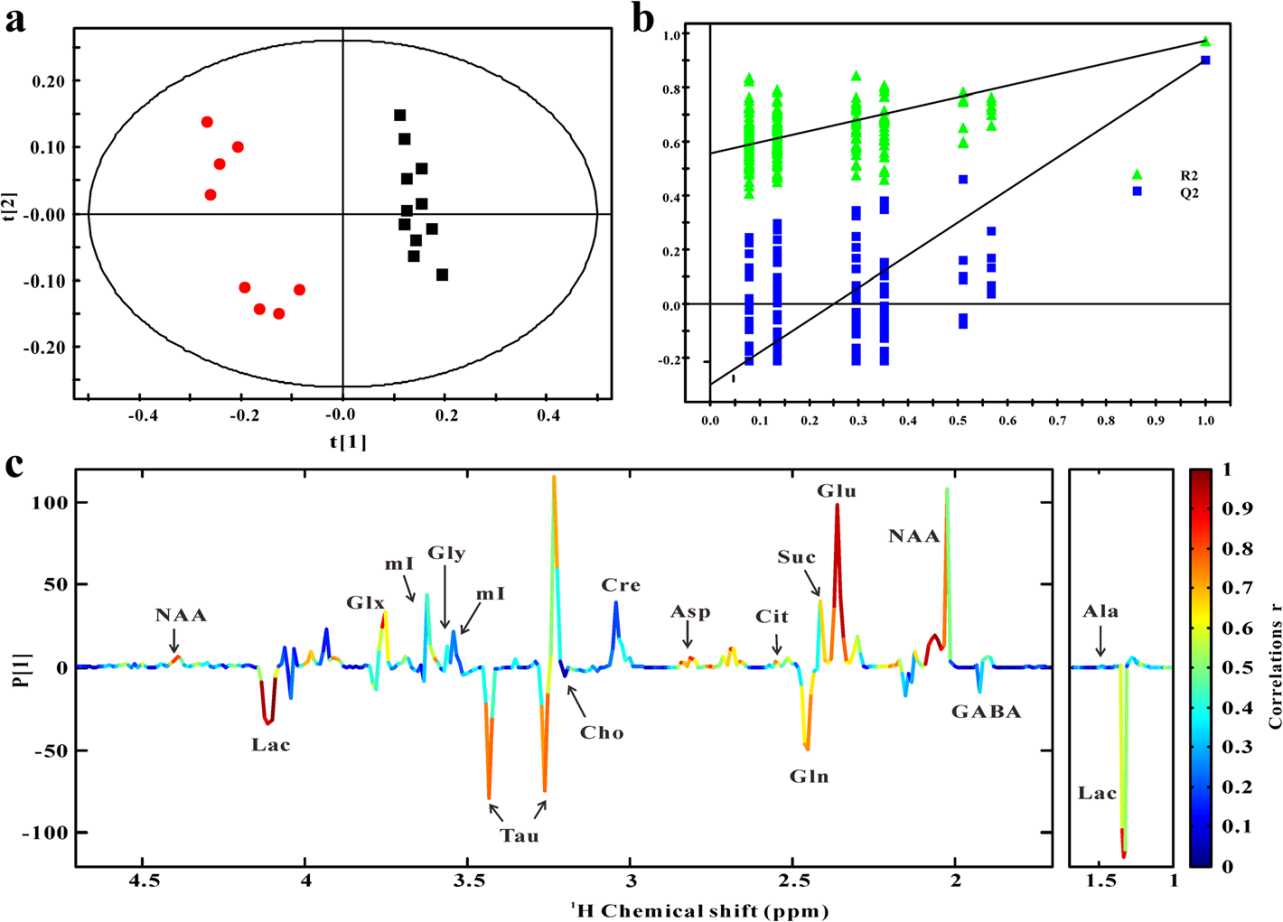
PLS-DA score plot (**a**) and validation plot (**b**) based on the ^1^H NMR spectra of hippocampus samples obtained from *db/db* mice (red circle, *n* = 7) and WT mice (■, *n* = 11). The coefficient-coded loading plot (**c**) corresponding to PLS-DA revealing the metabolites with large intensities responsible for the discrimination of the corresponding score plot

Representative ^1^H NMR-based metabolic profiling of the hippocampus extracts obtained from 17-wk *db/db* and WT mice was shown in Fig. 3. The spectral resonances of the metabolites assigned based on previous studies [19–21] and the 600 MHz library of the Chenomx NMR suite 7.0 (Chenomx Inc., Edmonton, Canada) were shown in Fig. 3b and Additional file 1: Table S1. Furthermore, projection to latent structure discriminant analysis (PLS-DA) was implemented to investigate the metabolic difference between *db/db* and WT mice (Fig. 4). As shown in Fig. 4a, clear discrimination was observed between them (R2X = 0.533, R2Y = 0.841, Q2Y = 0.585), which was validated by the permutation test (Fig. 4b). Figure 4c shows the corresponding loading plot with color-coded correlation coefficients (|r|) of PLS-DA, indicating metabolites that contributed to the separation between two groups. The results showed increased levels of lactate, glutamine and taurine, and decreased levels of glutamate, N-acetyl aspartate (NAA), citrate, glycine, choline, aspartate and succinate in hippocampus of *db/db* mice with DACD as compared with WT mice.

**Fig. 5:**
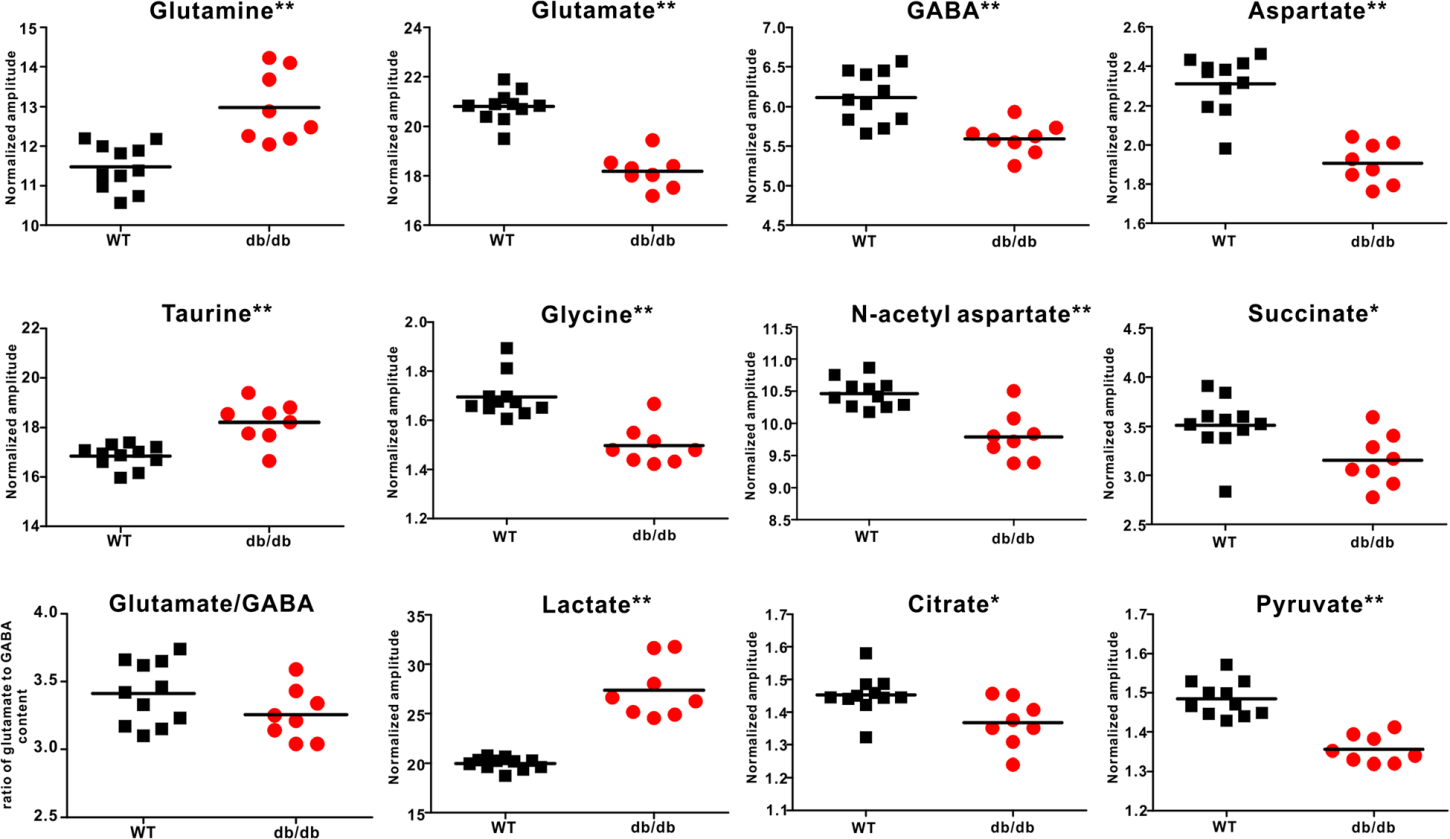
Changes in metabolite levels in the hippocampus of *db/db* mice with cognitive decline (red circle, *n* = 7) and WT mice (■, *n* = 11) obtained from NMR-based metabonomics. Significant level: ^*^ *P* < 0.05, ^**^ *p* < 0.01

To further confirm the metabolic changes, the levels of the metabolites were quantified and analyzed by t-test as shown in Fig. 5 and Additional file 1: Table S1. NAA level, a marker for neuronal viability, was decreased significantly in *db/db* mice with DACD (9.79 ± 0.37 vs 10.46 ± 0.22, *p* < 0.001). Lactate content in hippocampus of *db/db* mice with DACD was significantly increased (27.36 ± 2.89 vs 19.97 ± 0.60, *p* < 0.001). In addition, several tricarboxylic acid (TCA)-cycle-related metabolites, such as pyruvate, succinate and citrate, were also found significantly reduced in *db/db* mice with DACD. Taurine is a sulfur-containing amino acid that plays important roles on regulating osmolality of the astrocytes. In the present study, a significantly elevated level of taurine was observed in *db/db* mice with DACD (18.20 ± 0.83 vs 16.84 ± 0.45, *p* < 0.001).

Moreover, glutamate as an excitatory neurotransmitter (18.18 ± 0.68 vs 20.81 ± 0.63, *p* < 0.001) and GABA as an inhibitory neurotransmitter (5.59 ± 0.20 vs 6.11 ± 0.32, *p* < 0.001) were significantly reduced in hippocampus of *db/db* mice with DACD relative to WT mice. In contrast, as precursor and storage form of glutamate, the level of glutamine was elevated significantly (12.98 ± 0.89 vs 11.48 ± 0.57, *p* < 0.001). Thus, our data indicated the disturbance of the glutamate-glutamine cycle homeostasis in *db/db* mice with DACD.

### Key enzymes determination in glutamate-glutamine cycle

**Fig. 6:**
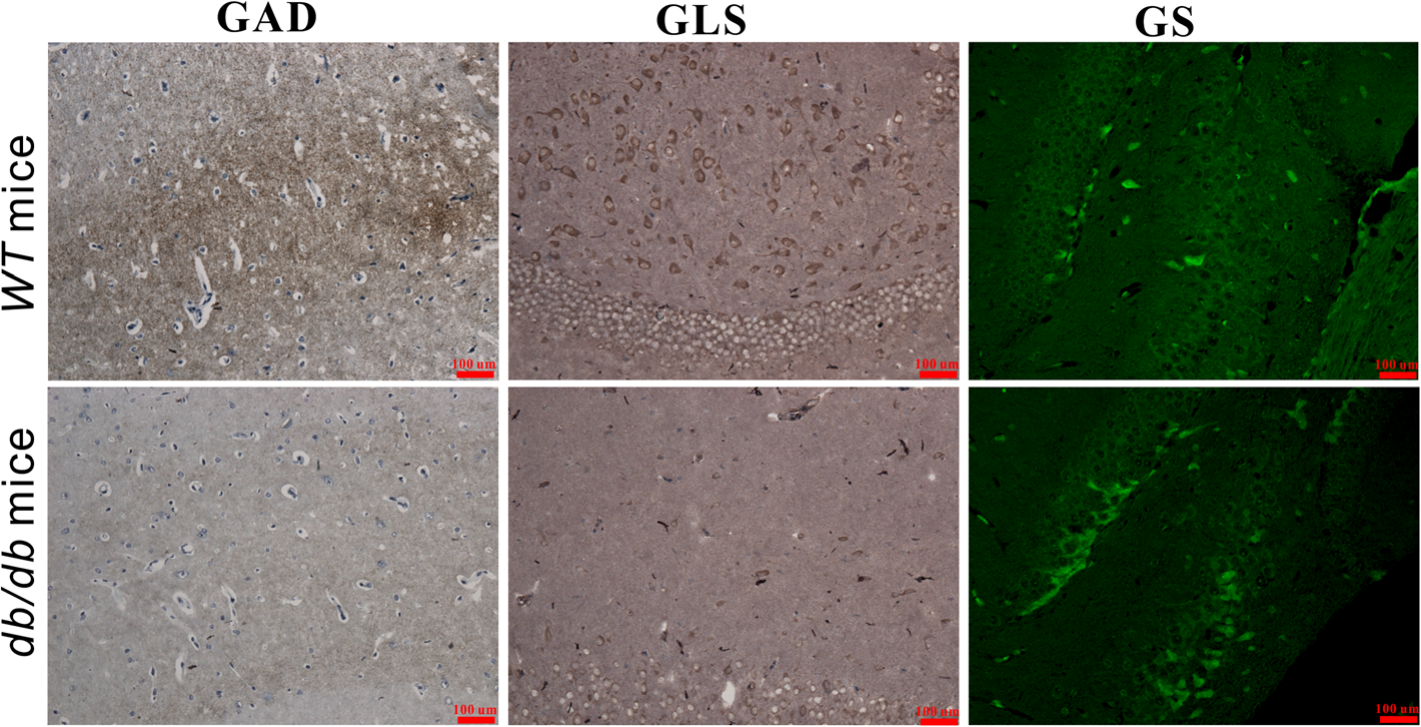
Immunohistochemistry of glutamic acid decarboxylase (GAD), glutaminase (GLS) and glutamine synthetase (GS) in the hippocampus of WT mice and *db/db* mice with cognitive decline

**Fig. 7:**
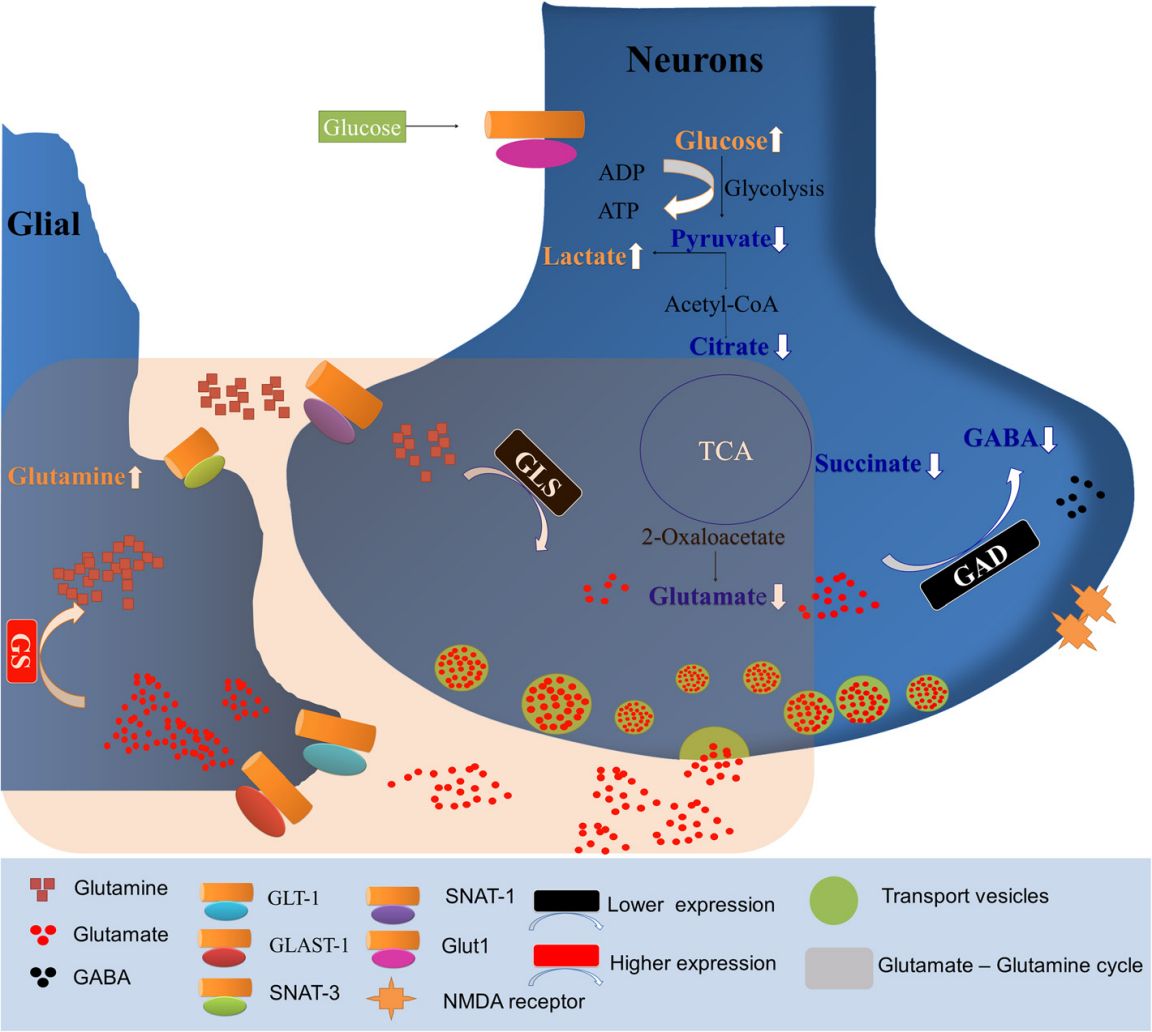
Summary of the metabolic changes in the hippocampus tissue of *db/db* mice with cognitive decline including glucose metabolism and glutamate–glutamine cycle compared with WT mice. In hyperglycemia circumstance, glycolysis is elevated, while aerobic oxidation is inhibited. As precursor for glutamate, reduction of TCA intermediates, combining with lower levels of glutaminase (GLS) and glutamate decarboxylase (GAD) in neuron, all contributes to the decreased level of glutamate and GABA. The reduced glutamate level, which is consistent with attenuation of postsynaptic glutamate receptors, *i.e.* N-methyl-D-aspartate (NMDA) subtype, and inhibition of synaptic long-term potentiation (LTP), may contribute to the pathogenesis of diabetes-associated cognitive decline. Keys: glutamate transporter 1 (GLT-1), glutamate aspartate transporter 1 (GLAST-1), sodium-coupled neutral amino acid transporters (SNATs), glucose transporter (Glut 1)

To further explore the reasons that glutamate-glutamine cycle influenced in *db/db* mice with DACD, we used immunohistochemistry and immunofluorescence to determine the alterations in some key enzymes involved in this cycle, such as glutamine synthetase (GS), glutaminase (GLS) and glutamate decarboxylase (GAD). GS, an ubiquitous enzyme present in the astroglial cytoplasm and involved in formation of glutamine from glutamate [22], was shown to be raised in hippocampus of *db/db* mice with DACD, which indicates an enhanced reaction from glutamate to glutamine (Fig. 6). Our data also show that immnuohistochemical labeling of GAD neurons [23] with a monoclonal GAD67 antibody revealed a decreased density of stained neurons, indicating that the pathway from glutamate to GABA was inhibited. In addition, a similar result was also shown in labeling with the anti-GLS antibody [24], which was consistent with the reduced trend from glutamine into glutamate. Figure 7 illustrates the metabolic changes in hippocampus of *db/db* mice with DACD relative to WT mice.

## Discussion

The present study was conducted to investigate the behavioral and metabolic changes in *db/db* mice with DACD. In the MWM test, we found that *db/db* mice had significantly longer latency to locate the hidden platform than WT mice. Moreover, number of crossing the original platform site was significantly reduced in *db/db* mice. The MWM test indicates cognitive ability was impaired in 17-week-old *db/db* mice. To explore potential mechanisms underlying DACD, we examined the change of cellular and metabolic levels by immunohistochemistry analysis and NMR-based metabonomics, respectively.

TUNEL is commonly applied to detect DNA fragmentation from apoptotic signaling cascades [25]. TUNEL assay in the present study shows an increase in apoptotic cells in hippocampus of *db/db* mice with DACD. Li et al. also found hippocampal neuronal apoptosis in rats with type 1 diabetes associated cognitive impairment [26]. In addition, GFAP is widely used as a specific marker of astrocytes [27]. In the present study, an increase in number of GFAP-positive cells in hippocampus of *db/db* mice with DACD compared with WT mice may suggest that astrocytes were proliferated in *db/db* mice with DACD, which is in good agreement with the previous work where they found that diabetes-induced memory impairment was accompanied by astrogliosis [28]. Therefore, neuronal apoptosis and astrocytes proliferation may be responsible for DACD in *db/db* mice.

Metabonomics reveals that DACD may be associated with energy metabolism disturbance. Since glucose is the main source of energy in the mammalian brain, regulation of glucose metabolism is critical for maintaining normal brain physiology [29]. Glucose metabolism is a catabolic process that firstly converts glucose to pyruvate, and then pyruvate is oxidized to CO_2_ and H_2_O under aerobic condition through TCA cycle or transformed into lactate by anaerobic glycolysis pathway. In the present study, a significant increase in lactate level may indicate that anaerobic glucose metabolism was enhanced in hippocampus of *db/db* mice with DACD, which was further confirmed by a decrease in several TCA intermediates, succinate and citrate. In addition, conversion of glucose to lactate can also occur in the presence of oxygen, which is known as the Warburg effect or aerobic glycolysis [30]. Thus, an increased lactate level may also indicate that DACD induced the Warburg effect in hippocampus of *db/db* mice.

Although glucose is the main brain energy substrate, glutamate is an important intermediate, linking the neurotransmitters metabolism and TCA cycle [31]. Glutamate homeostasis involves a mutual relationship between the neuron and neighboring astrocytes [32]. In the present study, DACD lead to reduced level of GLS and increased GS, which resulted in altered glutamate-glutamine cycle homeostasis in hippocampus of DACD mice. The glutamate-glutamine cycle is a major regulatory mechanism for fine tuning glutamate, glutamine and GABA levels in the organisms [33]. Lyoo et al. have found prefrontal altered glutamate-glutamine cycle in low cognitive performance patients with type 1 diabetes by using magnetic resonance imaging (MRI) [6]. However, due to overlapping of the chemical shift of glutamate, glutamine and GABA at 1.5-Tesla imaging, these signals were ascribed into only elevated glutamate which is a primary neurotransmitter. In the present study, using high-field-strength (14.09 Tesla) and tissue extraction technology, we can acquire more detailed information in chemical structure from an NMR spectrum. Thus, we found an increase in glutamine and a decrease in glutamate and GABA in *db/db* mice with DACD, which was in agreement with the findings from semi-quantitative immunohistochemistry about the key proteins alterations in glutamate-glutamine cycle. For instance, GS was strengthened in hippocampus of *db/db* mice with DACD, while both GLS and GAD were attenuated compared with WT mice. Thus, our results indicate that functional change of glutamatergic neuron in diabetes may be a possible reason underlying diabetes-related neurological complications. On the one hand, glutamate as an important neurotransmitter is involved in glutamate-glutamine cycle in brain [6]. On the other, glutamate is also known as a key molecule in the processes of learning and memory, which is released from the pre-synaptic nerve terminal and interacts with postsynaptic receptors, such as N-methyl-D-aspartate (NMDA) [6]. It is now well documented that the NMDA receptor expression is reduced in synaptic densities from the brain of chronic streptozotocin-induced type 1 diabetic rats [34] and other animal models with T2D [35]. However, the detailed relationship between DACD and the change in glutamate level and NMDA receptor activity remains unclear and needs further investigation.

## Conslusion

In the present study, we found that cognitive ability in *db/db* mice at 17-wk of age was impaired. To further explore potential mechanisms underlying this phenomenon, we investigated changes in cellular and metabolic levels using immunohistochemistry and NMR-based metabonomics, respectively. Results show that neuronal apoptosis and astrocytes proliferation may be responsible for DACD in *db/db* mice. In addition, metabonomics reveal that DACD development in *db/db* mice may be implicated in a reduction in energy metabolism and a disturbance of glutamate-glutamine cycle in hippocampus.

## Methods

### Animals

Male 15-wk *db/db* (BKS.Cg-m^+/+^ Leprdb/J, *n* = 11) and WT (C57BLKS/J-m^+/+^db, *n* = 15) mice were purchased from Mode Animal Research Center of Nanjing University. The mice were kept in a specific pathogen free colony of the Laboratory Animal Center of Wenzhou Medical University (Wenzhou, China) with regulated temperature and humidity and a 12:12-h light–dark cycle. The mice were fed with tap water and standard mice chow *ad labium* during the experiments. Body weight and fast blood glucose level was monitored weekly. The study was conducted in accordance to the “Guide for the Care and Use of Laboratory Animals” and approved by the Institutional Animal Care and Use Committee of Wenzhou Medical University (Document: wydw2012-0083).

### Morris water maze (MWM) test

The MWM test was performed according to a previously published method [36, 37], with a minor modification. Briefly, the test was conducted in a circular pool (diameter = 110 cm, height = 30 cm), filled with water made opaque with nontoxic paint and maintained at 26 ± 1 °C. The circular escape platform (diameter = 7 cm) was submerged 1 cm below the water surface. Cues were hung at four locations at the north, west, south, and east corners of the swimming pool wall, respectively. Four consecutive training days were performed, and on each-training day the mice swam four trials (rotating initial placement each time) for 60 s or until they located and climbed onto the hidden escape platform (within 60 s). The mice that failed to find the platform within 60 s were guided to be there by the operator. In addition, the mice were tested in a single 90 s probe trial without the platform at the last training day. The swimming path length, escape latency, and the swimming velocity were recorded by a computer system.

### Samples collection and ^1^H NMR spectra acquisition

The mice were sacrificed by decapitation at 17-wk of age, and specimens of hippocampus were dissected immediately, snap-frozen in liquid nitrogen and stored at −80 °C until use. Then the preparation of hippocampus extracts and acquisition of ^1^H-NMR spectra were performed using our previous method [17, 20, 21]. For NMR analysis, the hippocampus extracts were resuspended in 500 μL of D_2_O and centrifuged, and then the supernatant was transferred to a 5 mm NMR tube. ^1^H NMR spectra were acquired on a Bruker AVANCE III 600 MHz NMR spectrometer with a 5-mm TXI probe (Bruker BioSpin, Rheinstetten, Germany) at 25 °C. One-dimensional ZGPR pulse sequence was used, and the main parameters were set as follows: data points, 64 K; spectral width, 12,000 Hz; relaxation delay, 10 s.

### Multivariate pattern recognition analysis

The ^1^H NMR spectrum was phase- and baseline-corrected and integrated to binning data with a size of 0.01 ppm from 0.4 to 10.0 ppm using the Bruker Topspin 2.1 software package. For NMR spectra recorded in hippocampal extracts, the region of about δ 4.69–5.04 was removed to eliminate artifacts related to the residual water resonance. The remaining spectral segments were normalized to the total sum of the spectral intensity to compensate for variations in total sample volume. The normalized integral values were then subjected to multivariate pattern recognition analysis using the SIMCA-P+ V12.0 software package (Umetrics, Umea, Sweden). Data were visualized by the scores plots of the first two principal components to provide the 2D information [38].

The projection to latent structure discriminant analysis (PLS-DA), which is a supervised method, was carried out for class discrimination and biomarker identification. Data were visualized by plotting the scores of the first two principal components (PC1 and PC2) to provide the most efficient 2D representation of the information, where the position of each point along a given axis in the scores plot was influenced by variables in the same axis in the loading plot. PLS-DA revealed differences in the extracts composition of different groups, which were necessary to eliminate outliers and enhance the quality of the PCA model. The loading plots, which were assessed by the absolute value of the correlation coefficient, |r|, can identify the metabolites contribute to the separation of metabolic profiles [39]. A 100 random permutation test was performed to evaluate the robustness of the PLS-DA model. Meanwhile, two parameters were calculated: R2, the explained variance in the matrix, and Q2, the predictive capability of the model, which are commonly used to indicate the quality of model [40].

### Histology assay

Brain tissues were collected, fixed in PBS-buffered 10 % formalin for at least 24 h, and embedded in paraffin. The samples were then sectioned using a slicing machine (Leica, Germany) and mounted in Poly-L-Lysine-coated slides for histopathological examination. DNA fragmentation *in vivo* was detected by the one step TUNEL Apoptosis Assay KIT (Roche, Mannheim, Germany) and the images were captured with a Nikon ECLPSE 80i (Nikon, Japan).

For immunohistochemistry or immunofluorescence experiments, slides were placed in 1:20 citrate buffer in a decloaking chamber under pressure for 5 min after deparaffinizing, dehydrating and washing, depressurized for 5 min, and allowed to cool. Endogenous peroxidase was quenched using 15-min incubation in distilled water containing 0.3 % hydrogen peroxide. The transverse paraffin sections were incubated in 3 % H_2_O_2_ and 80 % carbinol for 30 min and then in blocking solution at room temperature for 1 h. Subsequently, the sections were incubated with the following primary antibodies overnight at 4 °C, including GFAP (1:1000, Abcam), GS (1:200, Santa Cruz), GAD65 (1:500, Abcam), and GLS (1:100, Abcam). After triple washing in PBS, the sections were incubated with horseradish peroxidase-conjugated secondary antibodies at 37 °C for 1 h. For immunohistochemistry, the reaction was stopped with 3,3’-diaminobenzidine and the image were captured at a magnification of 200 fold. For immunofluorescence staining, after primary antibody incubation, the sections were washed and then incubated for 1 h with secondary antibody (1:400) and the images were captured on Nikon ECLIPSE Ti microscope. The total number of TUNEL- and GFAP-positive neurons was manually counted from the five selected sections of the hippocampus.

### Statistical analysis

Difference analysis in identified metabolites between the DACD and WT mice was carried out by t-test using SPSS software (version 13.0, SPSS). *P* value of < 0.05 was considered statistically significant.

### Statement on ethics approval

The study was conducted in accordance to the “Guide for the Care and Use of Laboratory Animals” and approved by the Institutional Animal Care and Use Committee of Wenzhou Medical University (Document: wydw2012-0083).

### Consent for publication

Not Applicable.

### Data deposition

The data have been deposited to the KiMoSys repository (http://kimosys.org) [41] with the dataset identifier Data EntryID 91.

## Additional file

Additional file 1:
**Table S1.** Assignments of cerebral metabolites and their relative levels measured by ex vivo ^1^H NMR spectroscopy in hippocampus of mice. 1. p-value of DACD/control mice. (DOC 45 kb)

## Abbreviations

CNS: central nervous system
GABA: γ-aminobutyric acid
GAD: glutamate decarboxylase
GFAP: glial fibrillary acidic protein
GLS: glutaminase
GS: glutamine synthetase
MWM: Morris water maze
NAA: N-acetyl aspartate
NMR: nuclear magnetic resonance
TCA: tricarboxylic acid
