# Supplementary material for: Metabonomic profiles delineate potential role of glutamate-glutamine cycle in db/db mice with diabetes-associated cognitive decline
Source: Mol Brain. 2016 Apr 18;9:40. doi: 10.1186/s13041-016-0223-5 (PMC4835835; doi:10.1186/s13041-016-0223-5)
Supplement: Additional file 1: — Table S1. Assignments of cerebral metabolites and their relative levels measured by ex vivo 1H NMR spectroscopy in hippocampus of mice. 1. p-value of DACD/control mice. (DOC 45 kb) [file 13041_2016_223_MOESM1_ESM.doc]

**Additional file:**

**Additional Table S1.** Assignments of cerebral metabolites and their relative levels measured by ex vivo ^1^H NMR spectroscopy in hippocampus of WT and *db/db* mice.

| Metabolites | Integral (ppm) | Multiplicity | Control | DACD | P value |
| --- | --- | --- | --- | --- | --- |
| Glycine | δ3.54-3.56 | s | 1.69±0.08 | 1.50±0.08 | 0.00 |
| myo-Inositol | δ3.60-3.64 | dd | 12.58±0.50 | 12.20±0.65 | 0.18 |
| Taurine | δ3.40-3.44 | t | 16.84±0.45 | 18.20±0.83 | 0.00 |
| Choline | δ3.19-3.20 | s | 1.41±0.63 | 1.42±0.53 | 0.98 |
| Creatine | δ2.98-3.06 | s | 19.33±0.46 | 19.09±0.70 | 0.37 |
| Aspartate | δ2.80-2.82 | dd | 2.31±0.14 | 1.91±0.10 | 0.00 |
| Citrate | δ2.51-2.52 | d | 1.45±0.06 | 1.37±0.07 | 0.01 |
| Glutamine | δ2.45-2.50 | m | 11.48±0.57 | 12.98±0.89 | 0.00 |
| Succinate | δ2.39-2.41 | s | 3.51±0.28 | 3.16±0.27 | 0.01 |
| Glutamate | δ2.33-2.38 | m | 20.81±0.63 | 18.18±0.68 | 0.00 |
| Pyruvate | δ2.37-2.31 | s | 1.48±0.05 | 1.36±0.04 | 0.00 |
| GABA | δ2.27-2.31 | t | 6.11±0.32 | 5.59±0.20 | 0.00 |
| NAA | δ2.01-2.02 | s | 10.46±0.22 | 9.79±0.37 | 0.00 |
| Alanine | δ1.46-1.49 | d | 1.93±0.10 | 1.85±0.11 | 0.11 |
| Lactate | δ1.31-1.34 | d | 19.97±0.60 | 27.36±2.89 | 0.00 |
